# Building the adipose atlas: consensus guidelines and a standardized analytical workflow

**DOI:** 10.1093/lifemeta/loaf020

**Published:** 2025-06-09

**Authors:** Life Metabolism Editorial Team

Adipose tissue is a heterogeneous, dynamic, and metabolically active tissue that orchestrates systematic metabolic homeostasis by regulating thermogenesis, immune responses, and endocrine signaling [1–4]. Adipose tissue is a multicellular tissue with multiple cell types, including mature adipocytes, adipogenic stem and progenitor cells (ASPCs), immune cells, endothelial cells, and Schwann cells [5]. Dysregulation in adipose tissue is associated with a broad spectrum of metabolic disorders, such as obesity, type 2 diabetes, and cardiovascular disease [6, 7]. A comprehensive characterization of the cellular composition and functional states of adipose tissue is therefore essential for advancing the understanding of the physiological and pathological mechanisms underlying metabolic diseases. The advent of single-cell and single-nucleus transcriptomic (single-cell RNA sequencing [scRNA-seq] and single-nucleus RNA sequencing [snRNA-seq]) technologies has provided unprecedented resolution to tackle this challenge.

Nonetheless, adipose tissue presents unique and persistent obstacles to snRNA-seq analysis. The high lipid content, structural fragility, and pronounced depot-specific heterogeneity complicate tissue dissociation and nucleus isolation. Ambient RNA contamination and doublets arising from these experimental steps can introduce the diffuse expression of lipid-associated genes such as *PLIN1*, which can potentially lead to incorrect biological interpretations. In addition, one of the challenges in adipose tissue atlas research remains the collection and integration of data from multiple depots, disease states/stages, and physiological conditions, which are rarely feasible within a single laboratory. Consequently, consortium work from multiple labs becomes a general practice. As single-cell biology continues to expand into adipose tissue, there is a necessity for robust and standardized analytical frameworks to address the challenges.

In a recent study published in *Life Metabolism*, Dong *et al*. provide a technically optimized and benchmarked analytical pipeline specifically designed for the unique requirement of the analysis of snRNA-seq data in adipose tissues and other metabolically active tissues sourced from both human and murine models [8]. Meanwhile, a comprehensive review by the Human Cell Atlas (HCA) Adipose Bionetwork, led by Rosen, Mandrup, and colleagues, recently published in *Nature Metabolism*, provides a conceptual framework for the establishment of consensus adipose tissue atlases in both humans and mice [9]. These two publications mark a significant advancement in the single-cell characterization of adipose tissue.

The HCA Adipose Bionetwork review establishes a critical milestone by standardizing terminologies, sample collection protocols, and analytical steps within the field. This effort is indispensable, as disparate nomenclature, inconsistent marker usage, and diverse computational workflows have impeded cross-study comparisons. The authors propose a comprehensive framework for anatomical nomenclature across adipose depots as well as canonical marker genes for cell type annotation and suggest a similar analytical workflow as detailed in the publication by Dong and colleagues.

While the HCA Adipose Bionetwork review elucidates the “what” and “why” of the consensus adipose atlas, Dong *et al*. deliver the “how”. Their study introduced an end-to-end and reproducible computational pipeline for the analysis of snRNA-seq data from adipose tissue and other metabolic tissues. Now Dong *et al*. have deposited their entire workflow publicly available (metabomicslab.github.io/snRNAseq-analysis-workflow/), accompanied by well-documented tutorials and implementation scripts.

These two articles represent a pivotal alignment of conceptual clarity and computational precision in studying adipose tissue at single-cell resolution. The HCA Adipose Bionetwork review offers a cohesive structure, emphasizing the importance of analytical procedures. Dong *et al*. provide the executable, reproducible protocols that operationalize this vision. Beyond adipose tissue, the implications of the workflow by Dong *et al*. extend to other metabolically active tissues, including the liver, skeletal muscle, and hypothalamus, which encounter similar analytical challenges in scRNA-seq datasets. Dong *et al*. demonstrate that their computational strategy is scalable across these tissues, offering a generalizable framework for metabolic tissue analysis. As the field increasingly moves towards multi-organ and multi-modal approaches, this workflow will be advantageous to the community’s study of metabolic tissues as a whole.

Taken together, these two publications reinforce that high-quality single-cell research in adipose tissue depends not only on data collection and acquisition, but also substantially on data analysis and interpretation. As the field moves towards increasingly complex, multi-tissue, and multi-modal analyses, such foundational efforts are essential for ensuring precise interpretation of biological insights.
